# Pulmonary Metastasectomy versus Continued Active Monitoring in Colorectal Cancer (PulMiCC): a multicentre randomised clinical trial

**DOI:** 10.1186/s13063-019-3837-y

**Published:** 2019-12-12

**Authors:** Tom Treasure, Vern Farewell, Fergus Macbeth, Kathryn Monson, Norman R Williams, Chris Brew-Graves, Belinda Lees, Olivia Grigg, Lesley Fallowfield, PulMiCC Trial Group, Tom Treasure, Sion Barnard, Tim Batchelor, Chris Brew-Graves, Aman Coonar, Brian Davidson, Joel Dunning, John Edwards, Lesley Fallowfield, Vern Farewell, Simon Kendall, Olivia Grigg, Simon Grumett, Jurjees Hasan, Pauline Leonard, Belinda Lees, Eric Lim, Fergus Macbeth, Adrian Marchbank, Kathryn Monson, Apostolos Nakas, Ingrid Potyka, Mike Shackcloth, Bina Shah, David Tsang, Stelios Vakis, Norman R. Williams

**Affiliations:** 1 Clinical Operational Research Unit, University College London, London, WC1H 0BT UK; 2 MRC Biostatistics Unit, Cambridge, CB2 0SR UK; 3 Centre for Trials Research, Cardiff University, Cardiff, CF14 4Y UK; 4 Sussex Health Outcomes Research and Education in Cancer (SHORE-C), Falmer, BN1 9RX UK; 5 Surgical and Interventional Trials Unit (SITU), University College London, London, W1W 7JN UK; 6 Nuffield Department of Surgical Sciences, University of Oxford, Oxford, OX3 9DU UK

**Keywords:** Lung metastasectomy, Colorectal cancer, Randomised controlled trial

## Abstract

**Background:**

Lung metastasectomy in the treatment of advanced colorectal cancer has been widely adopted without good evidence of survival or palliative benefit. We aimed to test its effectiveness in a randomised controlled trial (RCT).

**Methods:**

Multidisciplinary teams in 13 hospitals recruited participants with potentially resectable lung metastases to a multicentre, two-arm RCT comparing active monitoring with or without metastasectomy. Other local or systemic treatments were decided by the local team. Randomisation was remote and stratified by site with minimisation for age, sex, primary cancer stage, interval since primary resection, prior liver involvement, the number of metastases, and carcinoembryonic antigen level. The central Trial Management Group were blind to patient allocation until completion of the analysis. Analysis was on intention to treat with a margin for non-inferiority of 10%.

**Results:**

Between December 2010 and December 2016, 65 participants were randomised. Characteristics were well-matched in the two arms and similar to those in reported studies: age 35 to 86 years (interquartile range (IQR) 60 to 74); primary resection IQR 16 to 35 months previously; stage at resection T1, 2 or 3 in 3, 8 and 46; N1 or N2 in 31 and 26; unknown in 8. Lung metastases 1 to 5 (median 2); 16/65 had previous liver metastases; carcinoembryonic antigen normal in 55/65. There were no other interventions in the first 6 months, no crossovers from control to treatment, and no treatment-related deaths or major adverse events. The Hazard ratio for death within 5 years, comparing metastasectomy with control, was 0.82 (95%CI 0.43, 1.56).

**Conclusions:**

Because of poor and worsening recruitment, the study was stopped. The small number of participants in the trial (*N* = 65) precludes a conclusive answer to the research question given the large overlap in the confidence intervals in the proportions still alive at all time points. A widely held belief is that the 5-year absolute survival benefit with metastasectomy is about 35%: 40% after metastasectomy compared to < 5% in controls. The estimated survival in this study was 38% (23–62%) for metastasectomy patients and 29% (16–52%) in the well-matched controls. That is the new and important finding of this RCT.

**Trial registration:**

ClinicalTrials.gov, ID: NCT01106261. Registered on 19 April 2010

## Background

Standard care of colorectal cancer patients includes detection by active surveillance of asymptomatic metastases followed by surgical resection in selected patients. Lung metastasectomy is now regarded as ‘a pillar of modern thoracic surgery’ [1] and is a substantial component of the work of thoracic surgical units internationally. This activity has been reported increasingly in clinical case series from the 1960s [2]. The publication of the International Registry of Lung Metastases in 1997 established lung metastasectomy in clinical practice [3]. The report contains data on patients who had had a lung metastasectomy performed by the contributing surgeons but, as is typical in procedure-based clinical reporting, there were no comparable data on those who did not have metastases removed. One small comparative study was published in 1980 [4]. It reported that the survival of 12 patients who were potential candidates for metastasectomy but did not have it was not dissimilar to 70 comparable patients who had had lung metastasectomy. The number of metastasectomy operations continued to increase during the period 2000 to 2011 [5, 6] without any randomised trials, a time when there were many controlled trials of systemic therapies [7]. In 2013 a meta-analysis of the 25 largest single-arm follow-up studies from 2000 to 2011, reported an overall 5-year survival rate of 41% for patients having lung metastasectomy for colorectal cancer, at an average interval of about 2 years after primary resection. No controlled studies were found and the authors concluded that ‘the benefit attributable to surgery is neither immediate nor irrefutable’ [8].

There is some indirect evidence from controlled trials that metastasectomy may not lengthen survival. There have been two meta-analyses of randomised trials comparing more with less intensive surveillance in patients treated for early colorectal cancer. Surveillance successfully advances detection: metastases were diagnosed up to 2 years earlier. There were more surgical interventions but there was no overall survival benefit [9, 10]. There was also uncertainty expressed by the authors of a meta-analysis of colorectal cancer survival gains who noted ‘that while indeed more metastasectomies are being performed, they have been made possible by better therapies and that this benefit should be ascribed to the therapies’ thus raising the possibility of reverse causation [7]. That is to say that longer survival provides opportunities for more treatments rather than additional treatments necessarily being the cause of longer survival.

Early expressions of doubt about the clinical effectiveness of lung metastasectomy pointed to the lack of control data [4, 11] but the weight of current opinion is that the observational evidence is sufficient [1]. However, the proposed criteria set out for trusting clinical observation, without the need for a controls, are not met [12]. The effect of the intervention has to be mechanistically plausible and a close temporal association between the intervention and the desired outcome is required. The variable course of cancer, and the deliberate selection of patients with very few metastases and a slower course, makes picking the signal from the noise impossible [12]. The missing evidence is control data on the survival of patients with features making them eligible for, but who did not actually have, metastasectomy. The need for this evidence led to the Pulmonary Metastasectomy in Colorectal Cancer (PulMiCC) randomised controlled trial which we report here.

## Methods

### Study design

PulMiCC was a two-stage, randomised, Phase-III, parallel-arm, multicentre trial.

The setting was hospital-based multidisciplinary teams (MDTs) managing patients with advanced colorectal cancer. The principal investigator (PI) at each trial site was a medical member of the team, either a surgeon or an oncologist. The study was set up in 24 hospitals treating advanced colorectal cancer: 21 were in Britain, with one each in Serbia, Italy and China.

The trial was co-ordinated from October 2009 to March 2014 by the Clinical Trials and Evaluation Unit, Royal Brompton and Harefield NHS Foundation Trust, London. PulMiCC administration and trial management then moved to the Surgical and Interventional Trials Unit (SITU), University College London. Both units are subsequently referred to as ‘the Trials Unit’. The co-ordination of patient-reported outcomes (PROs) throughout was at Sussex Health Outcomes Research and Education in Cancer (SHORE-C), University of Sussex.

### Ethics approval and consent to participate

Central ethical approval was confirmed from the National Research Ethics Committee London – Hampstead (ref approval no. 10/H0720/5) and did not begin recruiting at other centres in the trial until local ethical approval was obtained. Written informed consent was obtained at enrolment (Stage 1) and separately at randomisation (Stage 2).

The trial protocol can be accessed on line.

https://www.ucl.ac.uk/clinical-operational-research-unit/sites/clinical-operational-research-unit/files/pulmicc_protocol_december_2015.pdf

A description of the trial can be accessed on line. https://clinicaltrials.gov/ct2/show/study/NCT01106261?show_desc=Y#desc

### Patient participants

Eligible for inclusion were adults who had undergone resection of primary colorectal cancer with a prospect of cure, but who now had pulmonary metastasis, confirmed on routine review. In participating centres all such patients were reviewed by a properly constituted multidisciplinary team (MDT) responsible for all management decisions, advice and support of patients. Previously treated liver metastases were allowed but there had to be no other metastatic site. There had to be no clinical indication of active colorectal cancer, by investigations including imaging by computerised tomography (CT) and positron emission tomography (PET). Exclusion criteria were previous malignancy, concurrent illness, or unavailability for follow-up that was likely to interfere with treatment per protocol or the measurement of endpoints, or if mental incapacity precluded fully informed consent.

Biopsy proof was preferred but if, based on the above investigations, there was 90% clinical confidence that the diagnosis was of colorectal metastasis that was accepted.

Patient participants were recruited from the MDT meetings, invited to participate and registered for evaluation in Stage 1 after written informed consent. Those subsequently eligible for randomisation and for whom the MDT was in equipoise about the benefit of metastasectomy, were offered random assignment to have metastasectomy or not, after receiving a full explanation and giving written consent (Stage 2). All participants had continued active monitoring.

Trial process: a designated clinical team member informed potentially eligible patients of the MDT findings and explained the trial, emphasising the uncertainty of the evidence for the management of pulmonary metastases. Those interested in participating were given a patient information leaflet and an explanatory Digital Video Disc (DVD) to take home. A healthcare professional training DVD was also available for clinicians to aid their discussions with patients. The trial was administered by clinical trials staff at the local hospital site under the direction of the PI. Medical MDT members provided information and dates of events and measurements appropriate to their specialty. These were collated locally and returned to the Trials Unit on Clinical Report Forms (CRFs). Once a patient consented to join Stage 1, registration was carried out by the Trials Unit.

Following evaluation and any systemic treatments considered appropriate, eligible patients were approached by the oncologist or other designated member of the clinical team and asked if they were willing to consider the second (randomised) stage of the trial. All patients eligible for Stage 2 of the trial, whether or not they had chosen to proceed to randomisation, were invited to complete a questionnaire exploring their reasons for accepting or declining trials; this was completed at home and returned by post to SHORE-C [13].

Patients who confirmed their willingness to be randomised were asked to sign a second consent form and complete a set of baseline questionnaires: Functional Assessment of Cancer Therapy – General and Anaemia sub-scale (FACT-G-An) [14] plus selected items from the six-item Lung Cancer Brief Symptom Index [15] and the six-item short form of the Spielberger State/Trait Anxiety Inventory (STAI) [16]. The EQ-5D-3L (EuroQoL 5-Dimension 3-Level) questionnaire was administered for health economic evaluation [17].

### Randomisation and masking

Random assignment was to active monitoring (Control) or the same plus metastasectomy (Metastasectomy).

Randomisation was stratified by local site. Patients were allocated equally between the treatment arms. Sequence generation was at www.sealedenvelope.co.uk using a minimisation programme incorporating the participant’s age, sex, T(umour) stage, N(odal) stage, prior liver metastasectomy, time since resection of the colorectal primary cancer, number of metastases, and carcinoembryonic antigen (CEA) level. A random element was included such that each patient retained a non-zero probability of being randomised to each of the treatment arms. The trials staff transmitted the request and received the allocation electronically. Because the allocation was performed remotely, the process was completely concealed from the investigators and the Trials Unit.

Because the management options were so different (operation or no operation) blinding of participants and the site staff was not possible. The Trial Management Group (TMG) remained completely blind to allocation until after the primary analysis was done and the trial statistician and the TMG agreed the release of the full database for further analysis.

### Procedures

Control participants were to be managed without metastasectomy, radiotherapy or image-guided thermal ablation (IGTA). If these treatments were used subsequently, the patient remained in the assigned arm for follow-up at the specified time points from randomisation on the intention-to-treat principle.

Participants assigned to lung metastasectomy were to undergo surgery with the objective of an R0 resection (that is, histologically confirmed clear margins). The surgical approach (videothoracoscopy or open thoracotomy) was at the discretion of the surgeon.

Patients were seen for clinical examination including performance status, weight, lung function, CEA (carcinoembryonic antigen) assay and CT at 3, 6, 9, 12, 18, 24, 36, 48 and 60 months.

### Outcomes

The primary outcome was overall survival from the date of randomisation, with all patients being followed until the date of censoring or 60 months, whichever was shorter. Any surgery, radiotherapy, ablations or chemotherapy since the last report were recorded. In the event of crossovers a secondary analysis by treatment received was to be carried out.

Secondary endpoints were changes in lung function (forced expiratory volume in the first second (FEV1) and percentage predicted FEV1) and, over the period of 24 months following randomisation, patient-reported anxiety and quality of life.

The primary patient-reported outcome measure was the Functional Assessment of Cancer Therapy –Anaemia and Lung sub-scales (FACT An-L) Trial Outcome Index (TOI), which comprises the sum of scores from 37 items included in the FACT-G physical (seven items) and functional (seven items) well-being sub-scales together with the anaemia sub-scale (20 items) and three items from the Functional (Lung) Symptom Index Score (FLSI), not represented elsewhere in the FACT-G-An questionnaire [18–20].

### Statistical analysis

Sample size: a 10% difference in overall mortality at 3 years was taken to be the minimally important clinical difference (MID) and the inferiority margin for the design of the PulMiCC non-inferiority trial. Under the assumption of exponential survival curves, and with an expected 3-year survival rate of 30% in the interventional arm of the trial, then a 20% survival rate for the non-interventional arm would correspond to a relative risk of death for the non- interventional vs the interventional patients of 1.3. A sample size of 1350 registered patients was estimated to provide 1:1 randomisation of 300 patients. This was felt to be a practical sample size although it was hoped that perhaps as many as 150 additional patients could be randomised. Under the given survival assumptions, based on estimation of the log relative risk and the assumption that 72 patients from the pilot/feasibility study would be included with subsequent patients entering the trail uniformly over a 3-year period, simulations and asymptotic power calculations both indicated that a sample size of 300 would provide 78% power to detect an increased relative risk of death of 1.3 for patients in the non- interventional arm, when testing at the one-sided 5% level and this was adopted as the desired sample size in the trial protocol on this basis. This corresponds to a standard error of estimation for a 10% survival difference of 4.2%.

Comparative analysis: for the primary outcome of survival, Kaplan-Meier estimates of survival curves were produced. Treatment arms were compared through fitting a Cox relative-risk regression model [21], with an assumption of proportional hazards, which provided estimated hazard ratios and confidence intervals (CIs). The primary analysis was adjusted for minimisation variables. For the as-treated analysis, comparison was based on a time-dependent binary explanatory variable reflecting the time at which a metastasectomy occurred.

For lung function (FEV1 and predicted % FEV1) we used linear regression models with estimation using generalised estimating equations (GEE) to adjust for within-patient correlation. The primary analysis was to estimate a common effect of metastasectomy over time, with adjustment for follow-up time and baseline measurements, but variation in the treatment effect over time was examined. The potential impact of losses to follow-up was examined through fitting singular linear increment models [22]. Comparable linear model methods were used for patient-reported outcome data.

To reflect the high correlation between baseline and subsequent measures for all patient-reported outcomes (PROs) other than the STAI (anxiety), we used the change in scores from baseline at 3, 6, 12 and 24 months. For all outcomes, models which included baseline score were fitted to examine the appropriateness of differencing. Estimated differences between the treatment arms and associated 95% CIs were calculated and, where appropriate, compared with minimally important differences (MIDs). The null hypotheses addressed were of no differences in quality of life expected between Metastasectomy and Control. There was a particular focus on the alternatives that patients who are randomised to surgery would experience more lung function symptoms and that patients who are not randomised to surgery would experience greater anxiety.

## Results

**Fig. 1:**
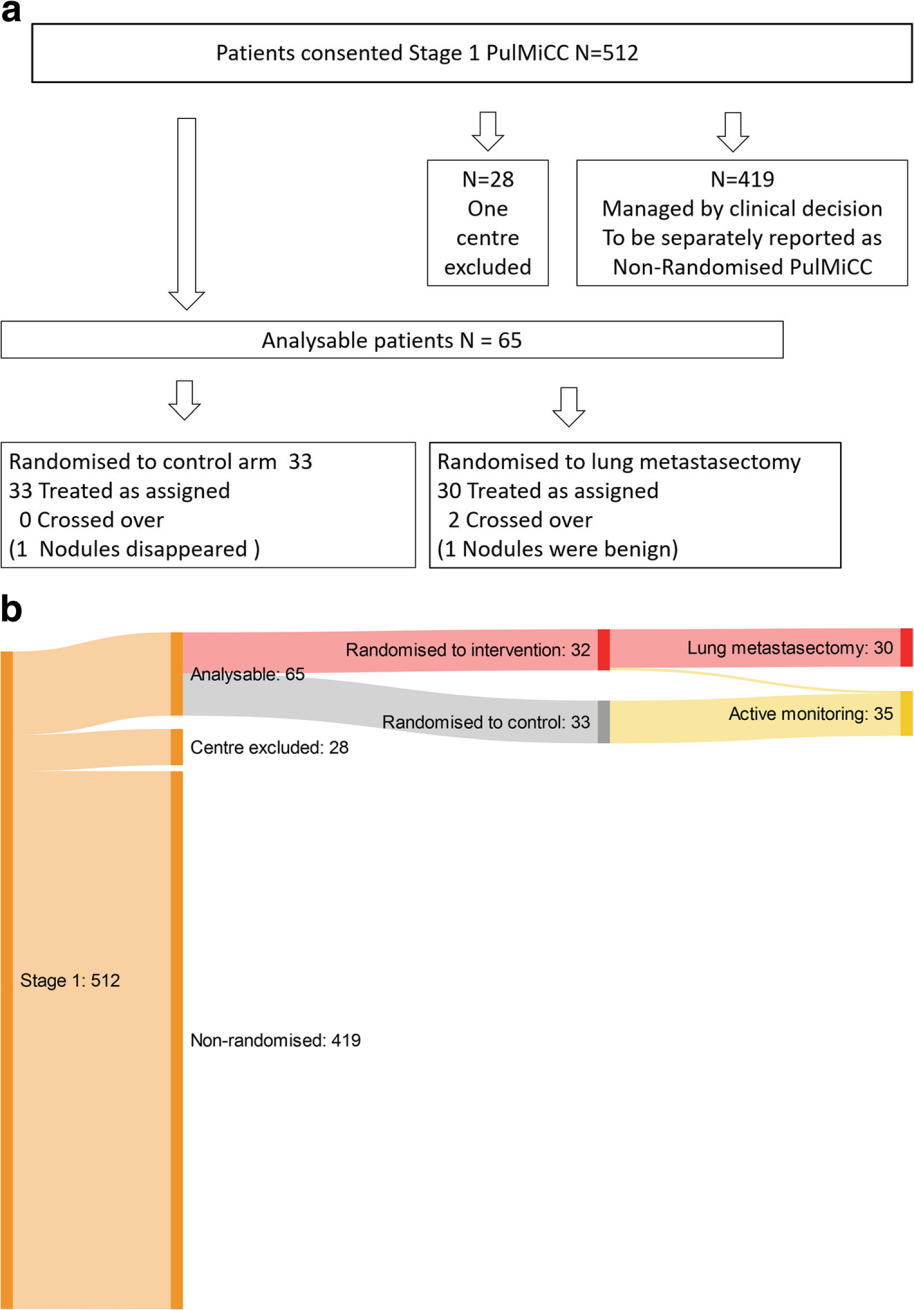
**a** The PulMiCC trial profile. **b** Sankey diagram of the PulMiCC trial flow through Stage 1, Stage 2, assignment and treatment

The first randomisation was on 2 December 2010 and the last on 24 November 2016. Recruitment slowed from 2015 and never recovered. We closed the trial in December 2016. At that time there were 512 of an intended 1350 registrations in Stage 1 and, of 300 patients required by the power calculation, we had randomised 93 (Fig. 1). The centre in Vojvodina, Serbia failed to return their CRFs because of unresolvable problems with trial support. We had only baseline data on two randomised patients, one in each group. After several discussions with the Independent Data Monitoring Committee (IDMC) we decided to exclude the site. Remaining from 13 sites, were 65 randomised participants, 33 in the Control arm and 32 assigned to Metastasectomy (Table 1). Apart from the excluded site in Serbia, no other patients have been lost from follow-up for the primary outcome which is survival. Ten sites registered patients but did not randomise any. The prospectively collected data on the full cohort of 512 patients and their survival will be analysed and reported separately as an observational study.

**Table 1:** Principle Investigators, Centre and the number of randomised

Principle investigator	Clinical centre	Randomisations
John Edwards	Sheffield Teaching Hospitals NHS Foundation Trust, Sheffield	18
David Tsang	Basildon and Thurrock University Hospitals NHS Foundation Trust**,** Basildon	8
Joel Dunning	The James Cook University Hospital, South Tees Hospitals NHS Foundation Trust, Middlesbrough	7
Mike Shackcloth	Liverpool Heart And Chest Hospital NHS Foundation Trust, Liverpool	7
Tim Batchelor	Bristol Royal Infirmary, University Hospitals Bristol NHS Foundation Trust, Bristol	5
Aman Coonar	Royal Papworth Hospital NHS Foundation Trust, Cambridge	5
Jurjees Hasan	The Christie NHS Foundation Trust, Manchester	4
Brian Davidson	Royal Free London NHS Foundation Trust, London	3
Adrian Marchbank	Derriford Hospital, University Hospitals Plymouth NHS Trust, Plymouth	2
Simon Grumett	New Cross Hospital, The Royal Wolverhampton NHS Trust, Wolverhampton	2
Eric Lim	Royal Brompton Hospital, Royal Brompton and Harefield NHS Foundation Trust, London	2
Apostolos Nakas	Glenfield Hospital, University Hospitals of Leicester NHS Trust, Leicester	1
Stelios Vakis	Queen’s Hospital, University Hospitals of Derby and Burton NHS Foundation Trust, Burton upon Trent	1
Total randomised		65

Minimisation produced balanced groups and limited the potential for unexpected confounding (Table 2).

**Table 2:** Variables used for minimisation. The process achieved well balanced groups

	Control (*N* = 33)	Metastasectomy (*N* = 32)
Male	20	21
Female	13	11
Age	Years	Years
Minimum	48	35
25%	61	61
Median	70	72
75%	76	74
Maximum	86	83
CRC T Stage		
T1	1	2
T2	4	4
T3	23	23
Missing	5	3
Total	33	32
CRC N Stage		
N1	15	16
N2	13	13
Missing	5	3
Prior liver resection		
Yes	9	8
No	24	24
CRC interval	Months	Months
Minimum	7.6	1.0
25%	17.4	13.9
Median	26.4	22.0
75%	34.8	36.8
Maximum	130.5	106.5
Lung metastases		
1	14	14
2 to 4	18	16
5 +	1	2

*CRC* colorectal cancer, *N* nodes, *T* tumour

**Fig. 2:**
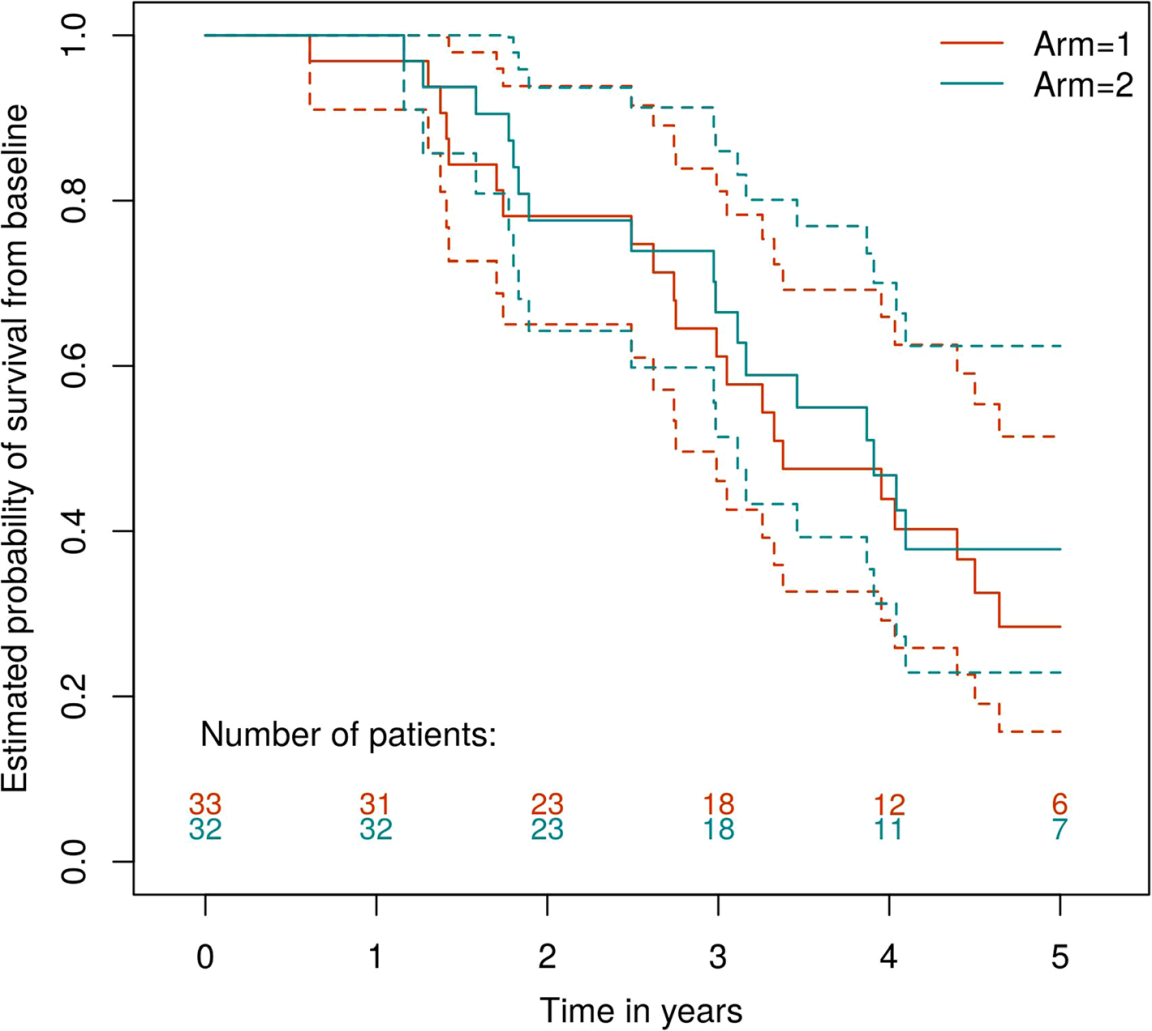
Kaplan-Meier analysis with 95% confidence intervals

Figure 2 presents estimated survival curves for the Metastasectomy and Control arms. There were 21 deaths in the Control arm and 17 in the surgical arm. The estimated hazard ratio comparing the relative survival rates in the Metastasectomy versus the Control treatment arm, adjusting for and, therefore, comparing patients with comparable minimisation variables, was 0.69 with a 95%CI of (0.35,1.37). The unadjusted estimated hazard ratio was 0.82, 95%CI (0.43, 1.56) and unadjusted non-parametric median estimates, in years, were 3.91, 95%CI (2.99,∞), and 3.38, 95%CI (3.11,∞), for the Metastasectomy and Control arms, respectively. Overall estimated survival at 4 years for the Control group was 40% (95%CI 26–63%) and 43% (95%CI 27–66%) for those assigned to metastasectomy. At 5 years, estimated survival was 29% (16–52%) and 38% (23–62%) for the Control and Metastasectomy arms. The 5-year gap (in estimated survival) emerges as there were 3/11 deaths in the Control arm in year 5 and 1/9 in surgery.

For the ‘as treated’ analyses, two patients assigned to metastasectomy did not undergo surgery, both of whom died. No patient in the Control group had crossed over to metastasectomy at the 3-month evaluation point and there was only one thereafter, 27 months after randomisation. The comparable adjusted and unadjusted estimated hazard ratios for these analyses were 0.60, 95%CI (0.30, 1.22) and 0.78, 95%CI (0.41, 1.50). Two patients turned out to not have colorectal lung metastases, one in each arm. The patient in the surgical arm had two intrapulmonary lymph nodes resected. The patient in the Control arm had three lung opacities which were not biopsied and disappeared spontaneously over subsequent months. They remain in the analysis based on intention-to-treat.

At 5-year follow-up FEV1-related measurements were only available for three patients in the Metastasectomy arm and one in the Control arm. This precludes informative analysis at this time point and treatment comparisons were, therefore, restricted to time points up to 48 months. Based only on observed patients, for FEV1 there is no evidence of an effect with an estimated average difference over time, based on GEE and adjusted for baseline and follow-up time, of − 0.05, 95%CI (− 0.21, 0.12). For percentage predicted FEV1, there is an estimated overall effect associated with metastasectomy of − 4.93, 95%CI (− 10.57, 0.70). The correlations of subsequent lung function measures with baseline are 0.855 and 0.75 for FEV1 and percentage predicted FEV1, respectively.

**Fig. 3:**
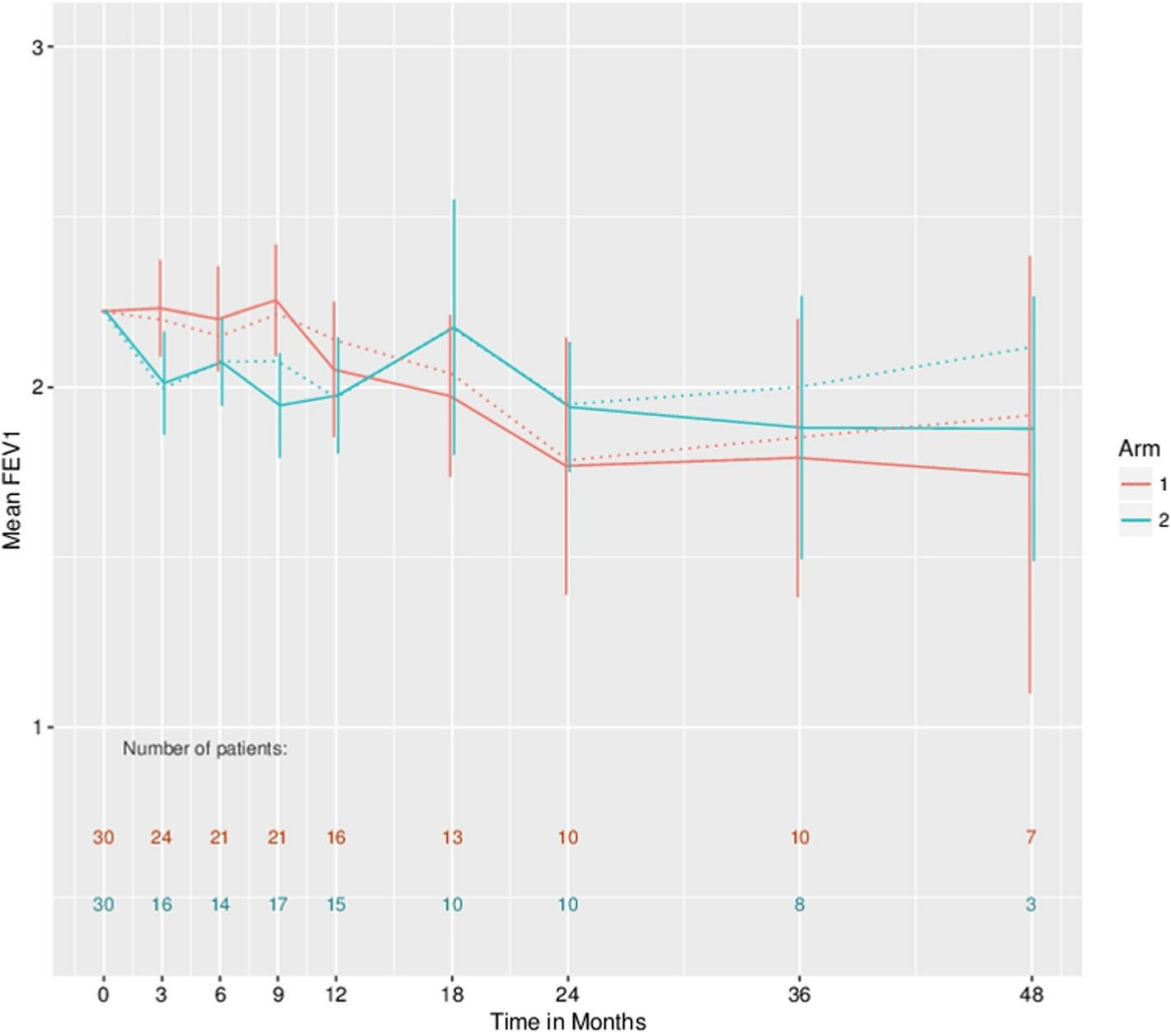
Estimated mean forced expiratory volume in the first second (FEV1) values in the two treatment arms with a common baseline starting value assumed in both arms corresponding to the average baseline in all patients. Dashed lines based on generalised estimating equations and solid lines based on singular linear models that adjust for drop-out. The 95% confidence intervals are provided for the singular linear model fits

**Fig. 4:**
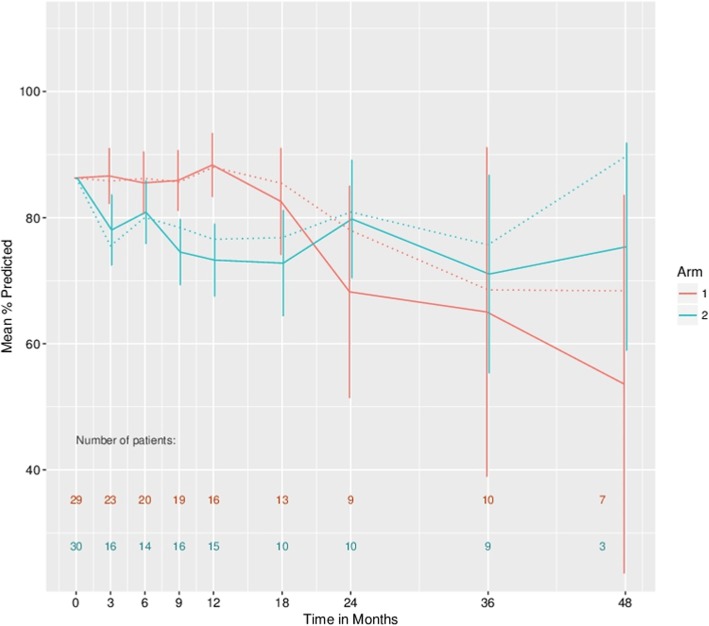
Estimated mean percentage predicted forced expiratory volume in the first second (FEV1) values in the two treatment arms with a common baseline starting value assumed in both arms corresponding to the average baseline in all patients. Dashed lines based on generalised estimating equations and solid lines based on singular linear models that adjust for drop-out. Confidence intervals are provided for the singular linear model fits

Figures 3 and 4 present estimates of the mean FEV1 and percentage predicted FEV1, respectively, in the two treatment arms at various follow-up times, with a common baseline starting value assumed in both arms corresponding to the average baseline in all patients. The means are connected by straight lines for presentation purposes only. Estimates based on singular linear models that illustrate the possible effect of drop-out are presented here. It can be seen that the apparent observed increase in FEV1 and percentage predicted FEV1 values at later follow-up times may be importantly influenced by drop-out. For both sets of estimates, there is an apparent cross-over of the values with the metastasectomy patients having lower values in the first year or so but showing an increase thereafter. Formal 1 df tests, based on GEE estimation, for variation in the arm effect over (linear) time generated *p* values of 0.11 and 0.02 for FEV1 and % predicted FEV1, respectively.

There were no treatment-related deaths or major adverse events.

A total of 21 of 65 patients were reported by CRF as treated with chemotherapy over the course of the 5 years with no significant difference in numbers between the two arms: Control 9/33; Metastasectomy 12/32. Some patients had received repeated treatments. Chemotherapy within 6 months of randomisation was given in five patients in each arm.

A total of 11 patients had had radiotherapy in the 5-year follow up period, 6/33 in the Control group and 5/32 in the Metastasectomy groups, none of which were within 6 months of randomisation and were dispersed without a pattern thereafter. In at least one instance in each group the reported radiotherapy was to treat metastases elsewhere (brain and bone). Two patients in each group were treated with radiofrequency ablation. No treatments were in the first 6 months after randomisation.

All but one patient had an ECOG (Eastern Cooperative Oncology Group) performance score of 0 or 1 at baseline. There is no suggestion of a difference between the arms.

**Fig. 5:**
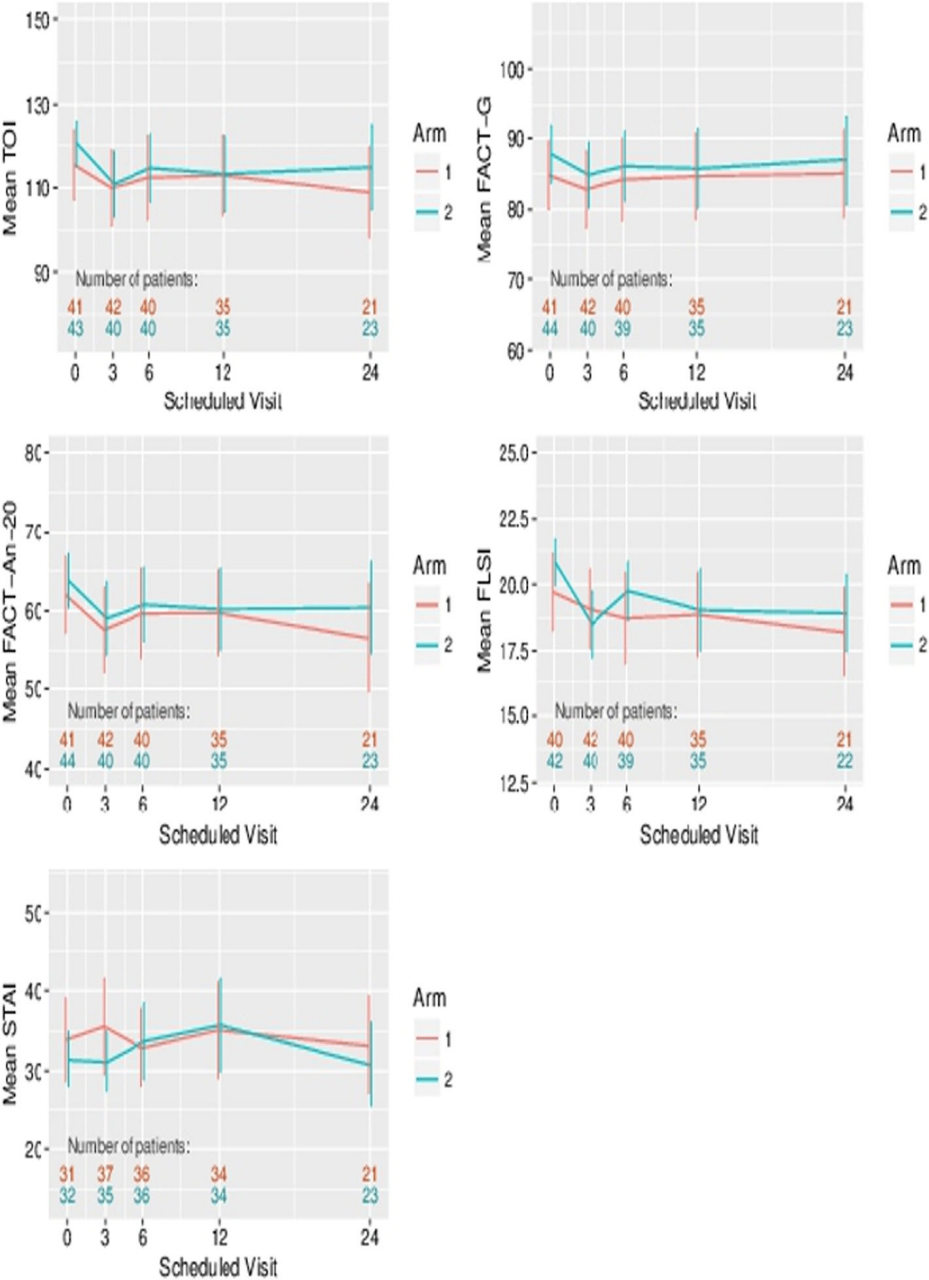
Patient-reported outcomes comparing the two arms of the trial. *TOI* Trial Outcome Index. *FACT-AnL* Functional Assessment of Cancer Therapy. *FACT-G* Functional Assessment of Cancer Therapy. General. *FACT-An-20* Functional Assessment of Cancer Therapy – Anaemia sub-scale. *STAI* Spielberger State/Trait Anxiety Inventory. *FLSI* Lung Cancer Brief Symptom Index

Figure 5 presents the mean patient-reported outcome scores over the 24 months of follow-up. For no outcome was a significant effect of metastasectomy detectable. For example, for the change in TOI from baseline, the estimated effect was − 1.51, 95%CI (−.90, 4.88). An early drop in the FLSI score (that is lung symptoms) for patients receiving surgery is observed as expected. While drop-out is significant, particularly at 24 months, linear increment analyses do not generate any qualitative difference for these outcomes. Minimal important differences for TOI, FACT-G, FACT-An-20 and FLSI, taken from the literature are 7.66, 5–7, 4.57 and 1.30, respectively. Such differences lie outside of, or towards the limit of the 95% CIs for the estimated effects for these four outcomes, on the change from baseline scale, which were (− 7.90,4.88), (− 5.77,3.02), (− 3.94, 4,45) and (− 1.56,0.56), respectively.

Variations in patients’ weights in the two arms of the trial was dominated by reducing numbers and widening standard deviation. There was no discernible difference between trial arms.

### Exploratory analysis of the reasons for not randomising

**Fig. 6:**
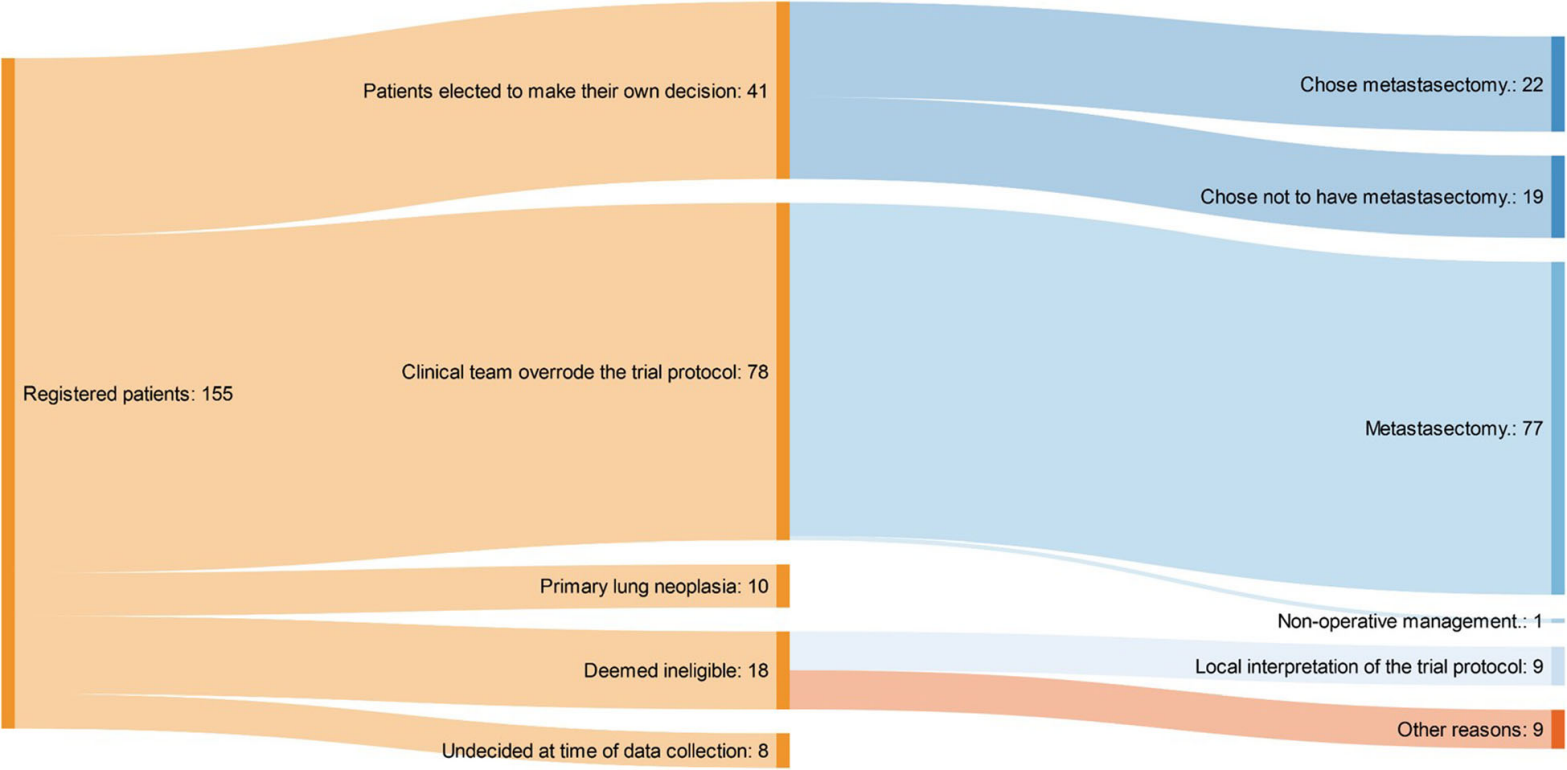
Sankey diagram of reasons for not randomising

The three most active centres (Sheffield, Liverpool and Bristol) were asked to provide reasons why patients consenting into Stage 1 of PulMiCC were not randomised. Of this subset of 155 patients, fully informed during the period of assessment, 41 made their own decision. The split to undergo or not undergo metastasectomy was 22:19. However, when the clinicians made the decision 99% (77/78) had metastasectomy. Ten patients had other pathology (nine lung cancer; one carcinoid). No constraint on the number of metastases was in the protocol but one unit set its own limits at two to four – deeming patients outside this range not eligible for randomisation. Of 18 patients deemed ineligible, half of the reasons were not aligned with the written protocol. At trial closure, of the 512 patients in Stage 1, 82% were not randomised resulting in an inconclusive result. Fig. 6 shows the Sankey diagram for reasons for non-randomisation.

## Discussion

Because of recruitment difficulties PulMiCC closed early and we were unable to reach the desired statistical endpoints and convincingly answer the question about the value of pulmonary metastasectomy. In the subset of 155 patients whose reasons for non-randomisation were examined, at least 56% were lost to randomisation by clinicians’ decisions. The 41 patients who decided for themselves whether or not to undergo metastasectomy did so in numbers which better reflected equipoise. Nevertheless, we believe that the results in 65 randomised patients have some important implications.

The survival of patients undergoing metastasectomy in PulMiCC was similar to that found in a quantitative synthesis of all follow-up studies up to 2007 [23] and the meta-analysis of larger observational studies up to 2011 [8]. Five-year survival was around 40% in all three. The PulMiCC 5-year survival (38% (23–62%)), therefore, appears to be a valid reflection of so called ‘real world’ practice. But survival of the PulMiCC Control patients was better than is generally assumed (29% (16–52%)) for those with untreated colorectal lung metastases. Because of small numbers, the confidence limits are wide but the difference between the survival of those undergoing metastasectomy and that of the untreated patients with colorectal lung metastases is likely to be smaller than is currently assumed. PulMiCC is the only randomised trial of colorectal cancer metastasectomy. Because the point estimate of the HR was 0.82, our findings are compatible with the belief that some patients, in whom lung metastases are truly the only residue of their colorectal cancer, may survive long term as a direct result of metastasectomy. But they call into question the belief that there is a very low likelihood of 5-year survival without metastasectomy in comparable patients.

Lung metastases generally remain asymptomatic and rarely contribute to terminal events and so there is unlikely to be significant palliative benefit from metastasectomy. There was a reduction in quality of life (QoL) at 3 months in those assigned to operation as would be expected among patients undergoing surgery (Fig. 5) and there was a detrimental effect on lung function from 3 months to 1–2 years after pulmonary resection compared with Control (Figs. 3 and 4). Neither difference was sustained or significant in the longer term, but the already small numbers of patients available for assessment declined by 3 years to fewer than 20 in each arm, most with ongoing cancer, making data uninterpretable. Although ‘psychological benefit’ is given as a justification for metastasectomy, we found no difference in anxiety between the two arms. Individual patients may have felt relieved to be rid of the radiologically visible vestiges of their cancer, but reduced anxiety was not seen as a group effect in this controlled trial. The lack of difference also indicates that informed patients can come to terms with the presence of lung metastases. This is in line with the only one late crossover from the Control to the Metastasectomy arm of the trial.

Comparative ‘before and after’ data on pulmonary function were not given in any of observational studies [2] which is consistent with the under-reporting of harms that has been found to be a feature of cancer trials [24]. The overriding limitation of this study is its small size with only 65 participants. This was, in large, part due to the difficulty clinicians had in presenting uncertainty to patients who were referred to them in the hope of cure [25]. It was also clear that the default of MDTs was to offer intervention rather than randomisation with a chance of assignment to a Non-metastasectomy arm. As a result of the subset analysis of 155 patients from the three most recruiting centres we think that this bias resulted in loss to randomisation of the majority of all patients who had consented to be in a randomised trial.

There are many well-documented instances where there has been a reversal from a prior standard of care after the fair test of a randomised controlled trial (RCT) [26, 27]. There are important precedents for finding that when subjected to a controlled trial, more radical surgery has not resulted in better cancer outcomes [28, 29]. Randomised trials of interventions, and particularly surgery versus no active treatment, are difficult to conduct and so tend to be relatively small but do provide a much more reliable estimate of differences between treatments than uncontrolled observations. More contentious are small trials which find no difference, such as the analysis of pooled trial data on 58 patients, which suggested that stereotactic radiotherapy might be have similar outcomes to lobectomy in the treatment of primary lung cancer [30]. PulMiCC is open to the same criticism of being small and, therefore, underpowered but if lung metastasectomy for colorectal cancer was not already in practice, it would not be possible to propose its introduction in the light of these findings. Although not proving an absence of any survival difference, a duty of candour should include sharing with patients that there might be no benefit from metastasectomy. That alone would help in recruiting to any future trials and improve evidence for clinical practice.

The difficulty faced by clinicians in declaring uncertainty is real and well recognised [31–35]. A trial which has been deemed ethically and scientifically sound should be presented to patients in a neutral and informative fashion by an individual trained and trusted to do this job. Clinical consultation is then about explaining the assigned treatment and about building trust and confidence. If the reverse occurs and the trial is first introduced by a clinical practitioner, it may be difficult to convey a sense of uncertainty and equipoise. In best clinical cancer practice the multidisciplinary team weighs up the options and then consultation is arranged with the appropriate treating clinician. In PulMiCC there was a clear exercise of bias with the MDTs overriding equipoise. This resulted in the exclusion of many patients who had given their informed consent. Learning from this and similar experiences, later UK trials of thoracic oncology (MARS-2, VIOLET) have recruited well after specific training in the QuinteT method for randomisation into surgical trials [35]. PulMiCC provides an example of the difficulties of running a randomised trial that challenges established clinical practice even when this is based on insecure observational evidence. It is easier to implement an intervention in the management of cancer, in the absence of evidence, than to seek the evidence that might demonstrate its futility.

The belief in metastasectomy is firmly entrenched; oligometastatic disease is now abbreviated to OMD [36–38]. The debate has moved on: it is not whether to treat, but how to treat. Should it be with surgery or IGTA including radiofrequency ablation and cryo-ablation? The current drive is towards stereotactic (ablative) body radiotherapy (abbreviated as SABR/SBRT) for metastases [39]. There has already been large investment; practitioners, for-profit health providers and the devices industry all expect a return on this investment [40]. However, it is probably more rational to treat systemic cancer with the now more effective systemic treatments [7].

In colorectal cancer the evidence from a meta-analysis of 16 RCTs showed no survival benefit from detecting metastases 1–2 years earlier, indicating that the growing practice of metastasectomy may not improve survival. These findings were regarded as ‘bleak nihilism’ by the *British Journal of Surgery’s* editor who wrote: ‘it is counterintuitive that earlier identification of metastatic disease does not improve survival’ [10]. The findings were confirmed by a Cochrane review [9]. The accumulated evidence from 16 RCTs is, for us, more persuasive than intuition. The retreat from radical mastectomy as the standard of care for breast cancer took many years of erosion of the intuition which pursued the belief that the bigger the operation the better [41]. Trials proved that it was time to call a halt to unavailing mutilation [28, 42].

The findings of PulMiCC should at least raise enough doubt for health services to call for better evidence, and it will require a larger number of randomised patients to show whether or not metastasectomy improves survival and, if so, by how much and for which patients. Five hundred and twelve patients consented to participate in the PulMiCC trial. Any future trial would have a power calculation informed by PulMiCC data but the implementation of the protocol would need to overcome the bias clearly exercised resulting failure to randomise such a high proportion of participants. Better training by methods such as QuinteT should be employed but it would also be important for clinicians with a vested interest in delivering particular treatments, including surgery, radiotherapy and other ablative techniques, to not be able to subvert the process of unbiased assignment, for reasons set out above [25]. In the light of the PulMiCC findings it seems improbable that the effect of excision or ablation of lung metastases can be as great as is believed at present.

## Data Availability

All information is freely available by application to the chief investigator TT and SITU UCL.

## Abbreviations

CEA: Carcinoembryonic antigen
CI: Confidence interval
CRC: Colorectal cancer
CRF: Clinical Report Form
CT: Computerised tomography
DVD: Digital Video Disc
ECOG: Eastern Cooperative Oncology Group
EQ-5D-3L: EuroQoL 5-Dimension 3-Level
FACT An-L: Functional Assessment of Cancer Therapy – Anaemia and Lung sub-scales
FACT-G-An: Functional Assessment of Cancer Therapy – General and Anaemia sub-scale
FEV1: Forced expiratory volume in the first second
FLSI: Functional Lung Symptom Index
GEE: Generalised estimating equations
IDMC: Independent Data Monitoring Committee
IGTA: Image-guided thermal ablation
IQR: Interquartile range
MDT: Multidisciplinary team
MID: Minimally important clinical difference
PET: Positron emission tomography
PI: Principle investigator
PRO: Patient-reported outcomes
PulMiCC: Pulmonary Metastasectomy in Colorectal Cancer
QoL: Quality of life
RCT: Randomised controlled trial
R0: No Residual disease histologically confirmed
SABR: Stereotactic ablative body radiotherapy
SBRT: Stereotactic body radiotherapy
SHORE-C: Sussex Health Outcomes Research and Education in Cancer
SITU: Surgical and Interventional Trials Unit
STAI: Spielberger State/Trait Anxiety Inventory
TMG: Trial Management Group
TNM: Tumour Nodal Metastasis staging system
TOI: Trial Outcome Index
